# In a team forgiveness climate, the influence of paradoxical thinking of leaders on the team voice behavior: Mediated by team cooperation

**DOI:** 10.1371/journal.pone.0265018

**Published:** 2022-03-15

**Authors:** Yijun Chen, Xu He, Lei Lu, Xiaoxiao Gao

**Affiliations:** 1 School of Business, Macau University of Science and Technology, Avenida Wai Long, Taipa, Macao, China; 2 College of Finance, Guizhou University of Commerce, Guiyang, China; Universita degli Studi di Perugia, ITALY

## Abstract

In order to clarify the influence of the paradoxical thinking of leaders on team voice behavior, a moderating mediation model was constructed to explore the mediating role of team cooperation and the moderating effect of team forgiveness climate based on the social exchange theory. Based on the "leader-employee" matching data of 477 employees from 101 teams, SPSS (Statistic Package for Social Science) and AMOS (a macro-micro model of Scotland) were used to analyze the three-stage data linear regression. The research conclusions indicate that: (1) The paradoxical thinking of leaders positively affects team voice behavior; (2) Team cooperation plays a completely mediating role in the relationship between the paradoxical thinking of leaders and team voice behavior; (3) The team forgiveness climate positively moderates the relationship between the paradoxical thinking of leaders and team cooperation, which means that their positive relationship is stronger in a higher forgiveness climate; (4) The team forgiveness climate moderates the mediating role of team cooperation between the paradoxical thinking of leaders and team voice behavior. Compared with a lower forgiveness climate, this moderating mediating effect is significant at a higher forgiveness level. This study clarifies the connection of the paradoxical thinking of leaders to the team voice behavior through team cooperation and has practical insights into how a team forgiveness climate promotes the team voice behavior.

## 1. Introduction

In order to adapt to the dynamic, competitive and complex market environment, team operation is favored. With intensified environmental uncertainty and changing demands, it is increasingly difficult to rely solely on the wisdom and management methods of team leaders to avoid organizational risks and improve organizational effectiveness [1]. Therefore, it becomes more relevant for the team to actively express their work-related views to achieve continuous team optimization in the incessant iterative process [1]. Positively team expression represents a new perspective on the behavior of suggestions, which is called the team voice behavior [2]. Van Dyne and LePine introduced voice behavior into organizational behavior [3]. Frazier and Bowler clearly defined team voice behavior as "the performance of all team members as a collated voice behavior" [3]. In China, research on team voice behavior is increasing day by day. In the available studies, Liu and Liao defined team prohibitive voice as "a working process or behavior that all team members express a common sense to the leader, that possibly impairs the team" [4]. In retrospect of existing global literature, team voice behavior can provide organizations with various ideas, opinions, and suggestions to help leaders become more informed of the work processes and problems, promote organization decision-making, and avoid error checking [5]. Team voice behavior can help prevent organizational risks, improve organizational effectiveness [6], and predict team performance [7].

As the core managers in the team, leaders can influence the team voice behavior with their seemly leading styles and behaviors [8–10]. Leaders can better manage the team with appropriate cognitive thinking [11–13]. Paradoxical thinking acts as a positive way of thinking that deals with contradictory things to discover new opportunities and experience new kinetic energy [12]. It conduces to integrate the two extremes of the contradiction, regards the tension and conflict caused by the paradox as an opportunity and challenge, and resolves disputes between contradictory needs, interests, ideas, and opinions. Consequently, the negative and tense working state of the team can be improved, and the positive expression can be stimulated, thus promoting team voice behavior. With the popularity of paradoxical leaders and gray management, the paradoxical thinking between leaders and employees in organizations will become the mainstream thinking mode advocated by enterprises [14]. However, most research focuses on the individual level of employees and leaders but rarely involves the team level [12,15–17] Similarly, most research on voice behavior focuses on employees. Research involving team voice only takes team characteristics as antecedent variables [18,19], without the influence of the thinking style of leaders on team voice behavior [8]. Previous studies have found that paradoxical thinking of leaders can improve team competitive advantage [20] and the good development of employees [21] and voice behavior [17,22]. However, the research of leadership paradoxical thinking on voice behavior at the team level is still relatively lacking. Therefore, in order to fill the gap in the research on the influence of paradoxical thinking of leaders on the team and to clarify whether the team voice behavior is affected by the leaders thinking, this study attempts to enrich and perfect the research related to paradoxical thinking and team voice behavior by analyzing the mechanism of paradoxical thinking of leaders affecting team voice behavior.

In addition, social exchange theory [23] suggests that after receiving benefits from others, individuals will provide relative benefits or help in return to maintain social exchange relationships. When team leaders or organizations offer support, care, and understanding to team members, team members perceive more responsibilities, obligations, and missions to repay the leader or the team, thereby expressing more positively and showing more voice behaviors. Leaders with a paradoxical thinking are adept in identifying, accepting, and proactively tolerating contrary tasks and requirements [24]. Faced with the paradoxical work requirements of accomplishing high-performance work and improving innovation capabilities, leaders with paradoxical thinking treat this contradictory work goal with an inclusive view [25]. They will be tolerant of team members by offering help even if the members do not complete their work. With the understanding and acceptance of the leaders, the team members show more communication, interaction, and cooperation. Team members work together to accomplish paradoxical works through mutual support and cooperation. Team cooperation and team voice behavior are also affected by the team atmosphere. An enabling team atmosphere promotes team cooperation to a certain extent, encourages team members to work towards common goals [26], and hearten employees to deliver suggestions and stimulate their work enthusiasm [27]. The core of social exchange theory is reciprocity [23]. In a team forgiveness climate, mistakes and failures of team members are tolerated in the face of paradoxical work [28–30]. In such a climate, to repay the tolerance and understanding, employees complete their work with mutual understanding, help, and cooperation by showing more proactive communication and expression.

Therefore, based on the social exchange theory, this research explores the mechanism of the paradoxical thinking of leaders on team voice behavior by adopting team cooperation as a mediating variable and team forgiveness climate as a moderating variable. First, this research starts with the thinking characteristics of team leaders and reveals the role of the paradoxical thinking features of leaders on team voice behavior. Second, from the perspective of social exchange theory, whether team cooperation is a bridge between paradoxical thinking of leaders and team voice behavior was tested, and the path by which the paradoxical thinking features of leaders influence on team voice behavior was revealed. Finally, from the perspective of mutual benefit, the moderating effect of a forgiveness climate on the relationship between the paradoxical thinking of leaders and team cooperation was explored in consideration of the contextual factors of team climate. Then, whether the contextual factors of the team forgiveness climate affect the paradoxical thinking of leaders was unveiled through the effect path of team cooperation. The above research is how this paper enriches the theoretical research on the formation of team voice behavior.

## 2. Research theory and hypothesis

### 2.1 Paradoxical thinking of leaders and team voice behavior

Team voice behavior refer to a concentrated expression of the suggestion behavior of team members [31]. This behavior includes interaction between group members, rather than a simple superposition of the suggestions of individual employees [32]. Frazier and Bowler clearly defined it as "the performance of all team members as a collated voice behavior" [33]. Team voice behavior helps the team avoid risks and improve organizational effectiveness and team performance [5–7].

In a work team, the team leader represents the core of the team. The personal thinking characteristics of a leader exert a huge impact on the team. As a stable trait tendency of the individual, the thinking mode can show continuous personal motivation and behavioral focus [34]. Leaders with paradoxical thinking are adept at proactively responding to and managing contradictions and conflicts. They tend to understand and accept team mistakes and failures using the connections between contradictory things [9]. According to the social exchange theory [23], pointed out that when the benefits provided by others are obtained, individuals will give relative benefits or help in return to maintain social exchange relationships. Team members who feel supported and encouraged by the tolerance and encouragement of leaders conduct more communication and discussion and become more proactively express their advice to repay the favorable behavior of leaders. Thus, the lack of information and wrong decisions of leaders in the management work can be avoided. First, while realizing organizational needs, leaders with paradoxical thinking also consider the individual needs of their teams. Allowing members to give full play to their strengths and abilities and carry out personalized authorization and decentralization can increase the work autonomy and diversity of the team members [35]. Employees will also give more suggestions in return to repay the leaders. Second, in an ever-changing organizational environment, leaders with paradoxical thinking can well-balance the competing needs in the organization. They are able to show their characteristics of openness, tolerance, and flexibility to help members feel an open and supportive environment where employees are more likely to deliver suggestions [36]. Finally, as the core of the team, the leader represents the role model for employees. Leaders with paradoxical thinking show the team how to deal with contradictory work in a complex environment. Faced with the possible risks of suggestions, team members will also imitate their leaders to consider the pros and cons of expressing suggestions and then choose the right time to do so. Hence, this research proposes Hypothesis 1:

**Hypothesis 1:** *The paradoxical thinking of leaders positively affects team voice behavior*.

### 2.2 The mediating role of team cooperation

The basis of team cooperation is the mutually perceived common goal among team members [37]. Team cooperation means that team members present a behavioral state of mutual dependence and support for the common goal within the team. Specifically, to achieve team goals, team members transform the output into results with certain cognitions and behaviors [38,39]. Thomas believes that team members have a win-win mentality for cooperation and that team cooperation makes it easier to achieve their own and team goals [40]. Studies have shown that when working cooperatively, team members are more willing to improve the performance of the team and the organization through mutual help [41]. When completing tasks cooperatively, they will show greater decision-making performance [42]. A team is more likely to succeed when its members cooperate with each other [43].

Team cooperation behavior is influenced by the leader and the team members themselves. Leaders act as role models in team cooperation, and their characteristic differences of thinking can affect the way team members work. First, leaders with paradoxical thinking characteristics integrate the two extremes of contradiction, showing more openness, support and guidance. According to the core view of social exchange theory—Mutual benefit and reciprocity [44], when leaders give more instructions for team work, team members will receive more support and guidance, thus deepening mutual understanding and cooperation among team members. Second, according to the social exchange theory, improve the relationship between leaders and employees, and between employees [45]. When team members experience the paradoxical thinking characteristics of the leader, it will be helpful to integrate conflicts and promote mutual understanding and help among team members, thus showing closer cooperation between them. Finally, leaders with paradoxical thinking are more likely to understand team failures or setbacks and display higher tolerance for team faults. According to the principle of mutual benefit and reciprocity in the basic social exchange theory [44], the understanding and tolerance of leaders enable team members to show more mutual communication, help, and cooperation to repay this favorable factor [46].

The work mode of team members lays the foundation of team voice behavior. First of all, team cooperation promotes close contact and communication between team members. Thus, voice behavior emerges when members express themselves proactively, facilitating the achievement of team organizational goals [26]. Secondly, leaders with paradoxical thinking characteristics set an example of understanding and tolerance. In addition, team members will imitate and show tolerance and understanding to others, prompting them to work toward common goals and actively offer advice and suggestions. Finally, team cooperation behaviors with clear team goals can promote better team development, strengthen the active expression of team members, and motivate team voice behavior. Therefore, this research proposes Hypothesis 2:

**Hypothesis 2:** *Team cooperation plays a mediating role in the paradoxical thinking of leaders and team voice behavior*.

### 2.3 The moderating role of forgiveness climate

Team forgiveness climate refers to the perception of team members being supported by the team when exhibiting benevolent and altruistic responses in the face of conflicts and failure [47]. It is mainly reflected in the tolerant attitude towards individuals who make mistakes or fail. Studies have shown that a forgiveness climate can promote the problem-solving efficiency of the work team [48], ease and improve the working relationship between team members [49]; it can also increase the productivity of employees, reduce turnover [50] and improve team performance [30,49].

The team climate is an important situational factor in team cooperation. In different team climates, even the same thinking characteristics or management style of the leader can produce various team behaviors. According to the social exchange theory, if an individual has previously been rewarded with a certain stimulus behavior, then when a similar one appears again, the individual may adopt the same behavior to reward others [51]. With the support and guidance given by the leader of paradoxical thinking, the team strives to achieve the team goals in the form of cooperation. At the same time, When the forgiveness climate releases a signal similar to the inclusion of failure, team members conduct more communication and cooperation to repay the forgiveness by teams, resulting in more proactive behaviors and expressions. Moreover, the team forgiveness climate is conducive to moderating the relationship between leaders and team members, improving the interpersonal relationship between team members, and forming a more friendly and trustworthy relationship. According to the social exchange theory, when faced with exchange risks, people will maintain new exchange relationships based on the trust established by previous exchange experience [51]. Therefore, when leaders with paradoxical thinking show understanding and acceptance in an inclusive and considerate team climate, team members will trust the leaders and team and cooperate more actively to complete team tasks and goals. Even if a short-term relationship crisis occurs between the team and the leader, they will tolerate and cooperate based on the original trust relationship. In addition, in a forgiveness team climate, team members tend to maintain an optimistic mood, no longer complain or blame others for their mistakes, at the same time, the team leader guides the work by integrating the thinking mode of contradiction. Promote staff to complete team work with good communication and cooperation [45,52]. On the contrary, members in a team with a low forgiveness climate, often regard failure as a shame, with intolerance of mistakes at work, shirk the work responsibilities. Even though the team leader with paradoxical thinking can integrate conflicts and contradictions, it is difficult for team members to communicate and cooperate to complete the work [46,53]. Thus, this research proposes Hypothesis 3:

**Hypothesis 3:** *The team forgiveness climate plays a positive role in the paradoxical thinking of leaders and team cooperation relationship*: *in a stronger forgiveness climate*, *the positive relationship between the paradoxical thinking of leaders and team cooperation is stronger*. *While in a weaker forgiveness climate*, *the positive relationship is weaker*.

After integrating the mediating role in Hypothesis 2 and the moderating role in Hypothesis 3, this study proposes a moderating mediation model. Team cooperation plays a mediating role between the paradoxical thinking of the leader and team voice behavior. However, this mediating role is affected by the degree of the team forgiveness climate. Specifically, when the team forgiveness climate is relatively high, the influence of the paradoxical thinking of leaders on team voice behavior can be transmitted through team cooperation. The team is infected by the forgiveness climate, and the acceptance and understanding of the paradoxical thinking of leaders play a role, enabling the team to cooperate and express themselves actively. Therefore, this research puts forward Hypothesis 4:

**Hypothesis 4:** *Team forgiveness climate positively moderates the indirect effect of paradoxical thinking of leaders through team cooperation that affects team voice behavior*: *a stronger team forgiveness climate indicates that this indirect effect is better*.

**Fig 1:**
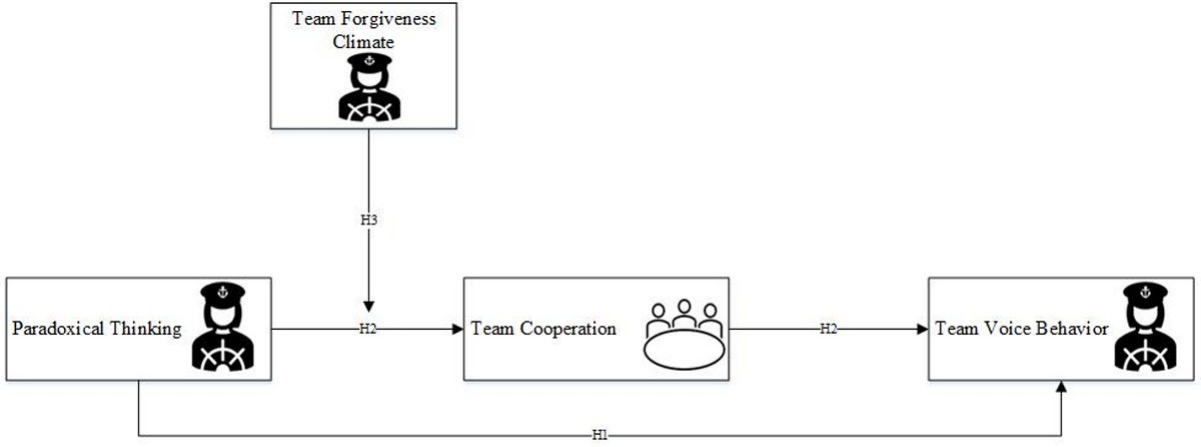
Research hypothesized model. Note: team member: 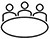 leader: 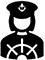.

In summary, based on the social exchange theory, this research takes Chinese mainland companies as research samples to explore the influence of the paradoxical thinking of leaders on team voice behavior. Moreover, based on theories and existing research findings, four hypotheses are put forward by introducing team cooperation as the mediating variable and the team forgiveness climate as the moderating variable. The specific research model diagram is shown in Fig 1.

## 3. Research design methods and variable measurement

### 3.1 Research samples and procedures

This study adopts questionnaire surveys to obtain reliable and realistic first-hand research data. The sample data is mainly acquired from enterprises in Shanghai, Guangdong, Zhejiang, Jiangsu, Guizhou, and Sichuan province in China. Data were obtained by a team of three people working jointly to distribute questionnaires, collect questionnaires, and unify the survey. Before the survey, leaders and team members in this study were informed that there was no right or wrong answer, with promised anonymity and confidentiality of the questionnaire. The "leader-employee" matching survey method was adopted. Data on paradoxical thinking, team forgiveness climate, and team voice behavior were answered by the leader, and team cooperation was answers by the team members. Moreover, in order to avoid common method deviations, this research was divided into three-time points to distribute questionnaires, with an interval of one-month, and a duration of 3 months (February to May 2021). The leader completed the first survey, including paradoxical thinking of leaders and a team forgiveness climate. A total of 150 questionnaires were issued, with 148 questionnaires returned. Team members completed the second survey, including team cooperation. A total of 560 employee questionnaires were distributed to 148 teams, with 510 employee questionnaires from 130 teams collected. In the third survey, leaders scored the team voice behavior. A total of 130 leader questionnaires were distributed, and 118 were returned. In addition, with the subjects’ permission, our research team added common control variables, including gender, ages, education level, working years, and team size.

After the completion of the survey, the last 4 digits of the mobile phone numbers were used as the matching basis for “leader-employee”. Finally, 477 valid questionnaires were obtained from 101 teams, with an effective team recovery rate of 67.33%. In the leader questionnaire, 62 are males, accounting for 61.4% of the survey, and 39 are females, occupying 38.6%; there are 19 people with college degrees, accounting for 18.8% of the survey, and 41 people with bachelor’ degrees, occupying 40.6%; 39 people have master’ degrees, with a proportion of 38.6%, and 2 people have doctor degrees, with a proportion of 2%. The average education level is at the undergraduate level, and the average ages are 40.09 years. The average working years are 11.78 years, and the average team size is 8.07. In the employee questionnaire, there are 217 males and 260 females, accounting for 45.5% and 54.5% of the survey, respectively. The average education level is at the college level, the average ages are 35.24 years, and the average working years with leaders are 5.54.

### 3.2 Measurement of variables

For the reliability and validity of the questionnaire, this study draws on the existing mature scales. Before the survey, according to a standard translation and back-translation procedure [54] and double-checking with the questionnaire distribution team, the scale was finally accurately translated into Chinese. This study adopts the Likert 5-point scale (1 to 5 in the questionnaire represent "strongly disagree" to "strongly agree", respectively).

#### Paradoxical thinking

The paradox-style scale compiled by Miron-Spektor et al. [12] was used to measure the paradoxical mindset of leaders. There are 9 items in the questionnaire. For examples, when dealing with conflicting views, I have a better understanding of the problem; "when trying to solve conflicting problems, I will be full of vitality." The Cronbach’s α coefficient of the scale in this study is 0.862.

#### Team forgiveness climate

With the forgiveness climate scale developed by Cox [48], there are 4 items in total. For examples, "in the team, we can tolerate the faults and mistakes of the team members; in the team, we do not hold grudges". The Cronbach’s α coefficient of the scale in this study is 0.845.

#### Team cooperation

Using the team cooperation scale compiled by Chatman and Flynn [55], there are 4 items in total. For examples, "It is important to maintain harmony within our team; there is a high degree of cooperation among our team members". The Cronbach’s α coefficient of the scale in this study is 0.838.

#### Team voice behavior

Based on the method suggested by the measurement team of Walumbwa et al. [56], Van Dyne and LePine’s [3] scale was adapted to measure individual-level suggestions, with a total of 6 items. For examples, "the team member comes up with and puts forward opinions and suggestions on problems that affect the work team; team members communicate with each other about their work, even if they have different opinions." The Cronbach’s α coefficient of the scale in this study is 0.801.

#### Control variables

Demographic variables have been found to influence voice behavior. Team voice behavior is affected by gender, age, length of service, and education level of the leader [12]. In addition, team size may impact the results of the study [57–59]. In order to verify the model more accurately, this study measures gender, age, length of service, education level of leaders, and team size as control variables.

## 4. Results

### 4.1 Common method bias analysis

Although this study adopts a multi-stage and multi-source research method to avoid homologous deviation in operation, paradoxical thinking, team forgiveness climate, and team voice behavior are all evaluated and answered by leaders. Therefore, a common method bias is necessary for the part of the questionnaire filled by leaders. Harman single factor test [60] was used for factor analysis of all problems. It is found that the variance explanation of the first factor is 34.29%, less than the recommended value of 50% [61]. Therefore, the relationship between variables is credible without obvious homology deviation. The overall research results are not be seriously affected.

### 4.2 Data aggregation test

When data at the individual level is aggregated to the group level, a consistency test of scoring is required [62]. Since this research focuses on the concept of team cooperation at a team level, the aggregation principle of “consistency coefficient R_wg_ not less than 0.70” is followed [63]. The results show that the average R_wg_ of team cooperation consistency is 0.938 (median R_wg_ is 0.963, minimum R_wg_ is 0.78, and maximum R_wg_ is 1.00). In addition, scholars have further pointed out that only when the intraclass correlation coefficient (ICC (1)) is greater than 0.12 with ICC (2) over 0.60 [63] can individual-level data be aggregated into group-level data. The result shows that the ICC (1) value of team cooperation is 0.143, and the ICC (2) value is 0.611. By combining the indicators of R_wg_ and ICC the score consistency test in this study meets the standard for data aggregation.

### 4.3 Discriminant validity test

In order to test the discriminative validity of the variables involved in this study, the structural equation model was used to carry out factor analysis to test the variables in the study. The team cooperation variables are aggregated to the team level after employees fill in, while paradoxical thinking, team forgiveness climate, and team voice behaviors are filled out by leaders. Team cooperation has been distinguished from other variables in the operation means of the research method. Therefore, this study only needs to verify the variables answered by leaders: the discriminative validity of paradoxical thinking, team forgiveness climate, and team voice behavior. Confirmatory factor analysis was used to test the discriminative validity of each variable [60]. Therefore, the fitting index was selected to judge the fitting degree of the model. The chi-square difference must reach the significant level, the root mean square of approximate error (RMSEA) must be less than 0.08, and the comparative fitness index (CFI) and Tuck-Lewis index (TLI) must be greater than 0.9. According to Table 1, the three-factor model is better than other factor models in the fit of the sample data (χ2 = 187.07, df = 120, RMSEA = 0.07, SRMR = 0.08, CFI = 0.93, TLI = 0.91). It is indicated that the discriminative validity of the questionnaire design in this study is sound, and three factors represent three different constructs that can be used for regression analysis.

**Table 1:** Results of confirmatory factor analysis (N = 101).

Model	χ2	df	Δχ2	RMSEA	SRMR	CFI	TLI
Three-factor model	187.07	120		0.07	0.08	0.93	0.91
Two-factor model (A+B)	252.79	122	65.72***	0.10	0.10	0.87	0.82
One factor model (A+B+C)	351.07	123	164***	0.14	0.13	0.78	0.69

A: Paradoxical thinking; B: Forgiveness climate; C: Team voice; “+” means integration.

### 4.4 Descriptive statistics and correlation analysis

To further clarify the relationship between the paradoxical thinking of leaders, team forgiveness climate, team cooperation, and team voice behavior, this research conducted a correlation analysis on the relationship between various variables. The results in Table 2 indicate that the correlation coefficients between variables in the hypothetical relationship are significant. This result also supports the validation of research hypotheses, but further verification is required.

**Table 2:** The mean, standard deviation and correlation coefficient of variables (N = 101).

Variable	Mean	Standard deviation	1	2	3	4	5	6	7	8
1 Gender	0.386	0.489								
2 Age	40.089	7.604	-0.176							
3 Education level	16.683	1.334	0.128	.209*						
4 Length of Service	10.198	6.198	-0.157	.738**	-0.030					
5 Team size	8.069	3.756	-0.058	-0.106	.258**	-0.168				
6 Paradoxical thinking	3.990	0.531	-0.131	-.228*	-0.050	-.032	.347***			
7 Forgiveness climate	4.253	0.583	0.014	-.224*	0.091	-.250*	.295**	.512***		
8 Team cooperation	4.131	0.286	-.206*	-.122	.028	-.076	.369***	.527***	.513***	
9 Team Voice	4.158	0.477	.214*	-.169	0.135	-.191	.358***	.286**	.473***	.398***

Note

***p<0.001

**p<0.01

*p<0.05.

### 4.5 Regression analysis

This study uses multiple linear regression and the process method to analyze the relationship among the paradoxical thinking of leaders, team forgiveness climate, team cooperation and team voice behavior. Among them, gender, age, and length of service of leaders, and team size are used as control variables. The results are shown in Table 3.

**Table 3:** Regression analysis model.

Variable	Team cooperation			Team voice		
	Model one	Model two	Model three	Model four	Model five	Model six
**Constant term**	4.199***(.183)	3.077***(.293)	2.671***(.284)	3.882***(.304)	2.902***(.525)	1.176*(.538)
**Control variables**						
Gender	-.119*(.055)	-.083(.050)	-.096*(.046)	.211*(.091)	.242**(.090)	.289**(.088)
Age	-.007(.005)	.001(.005)	.003(.005)	-.003(.009)	.004(.009)	.003(.009)
Length of service	.004(.006)	-.003(.006)	.000(.006)	-.005(.011)	-.011(.011)	-.009(.010)
Team size	.027***(.007)	.015*(.007)	.010(006)	.045***(.012)	.035**(.012)	.026*(.012)
**Variable**						
Paradoxical thinking		.239***(.051)	.115*(.055)		.209*(.092)	.075(.098)
**Mediating variable**						
Team cooperation						.561**(.176)
**Moderating variable**						
Forgiveness climate			.187***(.047)			
**Interactive item**						
Paradoxical thinking * Forgiveness climate			.214**(.078)			
F	5.514	9.711	11.03	5.709	5.806	7.008
R^2^	0.188	0.338	0.454	0.193	0.235	0.309
ΔR^2^	.188***	.151***	.266***	.193***	.041*	.116**

Analysis of control variables: According to the results of Model 1 in Table 3, compared with female-led teams, male-led teams are more inclined to team cooperation (B = -0.119, SE = 0.055, P < 0.05). In addition, in larger teams, members are more inclined to team cooperation (B = 0.027, SE = 0.007, P < 0.001). The results of Model 4 in Table 3 reveal that members show more team voice behavior in female-led teams (B = 0.211, SE = 0.091, P < 0.05) compared with that in male-led teams. Additionally, in larger teams, members are more apt to team voice behavior (B = 0.045, SE = 0.012, P < 0.001).

Analysis of Hypotheses 1 and 2: The results of Model 5 in Table 3 indicate that after controlling the natural attributes of the leader and team size, the paradoxical thinking of the leader positively affects the team voice behavior (B = 0.209, SE = 0.092, P < 0.05). Therefore, Hypothesis 1 holds. From the results of Model 6 in Table 3, it can be known that after controlling the natural attributes of the leader and team size, team cooperation has a mediating effect on the relationship between the paradoxical thinking of the leader and team voice behavior (B = 0.561, SE = 0.176, P < 0.01). In order to further clarify the above effect, the Bootstrap method was used for testing, and the results are shown in Table 4. After controlling the natural attributes of leaders and the team size, the indirect effect of team cooperation is significant with 0 excluded in the 95% confidence interval; the direct effect of the paradoxical thinking of leaders on team voice behavior is not significant, and the confidence interval of 95% is over 0. It is indicated that team cooperation plays a complete mediating role in the relationship between the paradoxical thinking of leaders and team voice behavior. Therefore, Hypothesis 2 is established.

**Table 4:** Bootstrap test of mediating effect.

	Effect size	Standard deviation	95% confidence interval	
			Lower confidence limit	Higher confidence limit
Indirect effect	0.134	0.060	0.029	0.262
Direct effect	0.075	0.098	-.119	0.269

Note: The sample size of Bootstrap is N = 5000.

**Fig 2:**
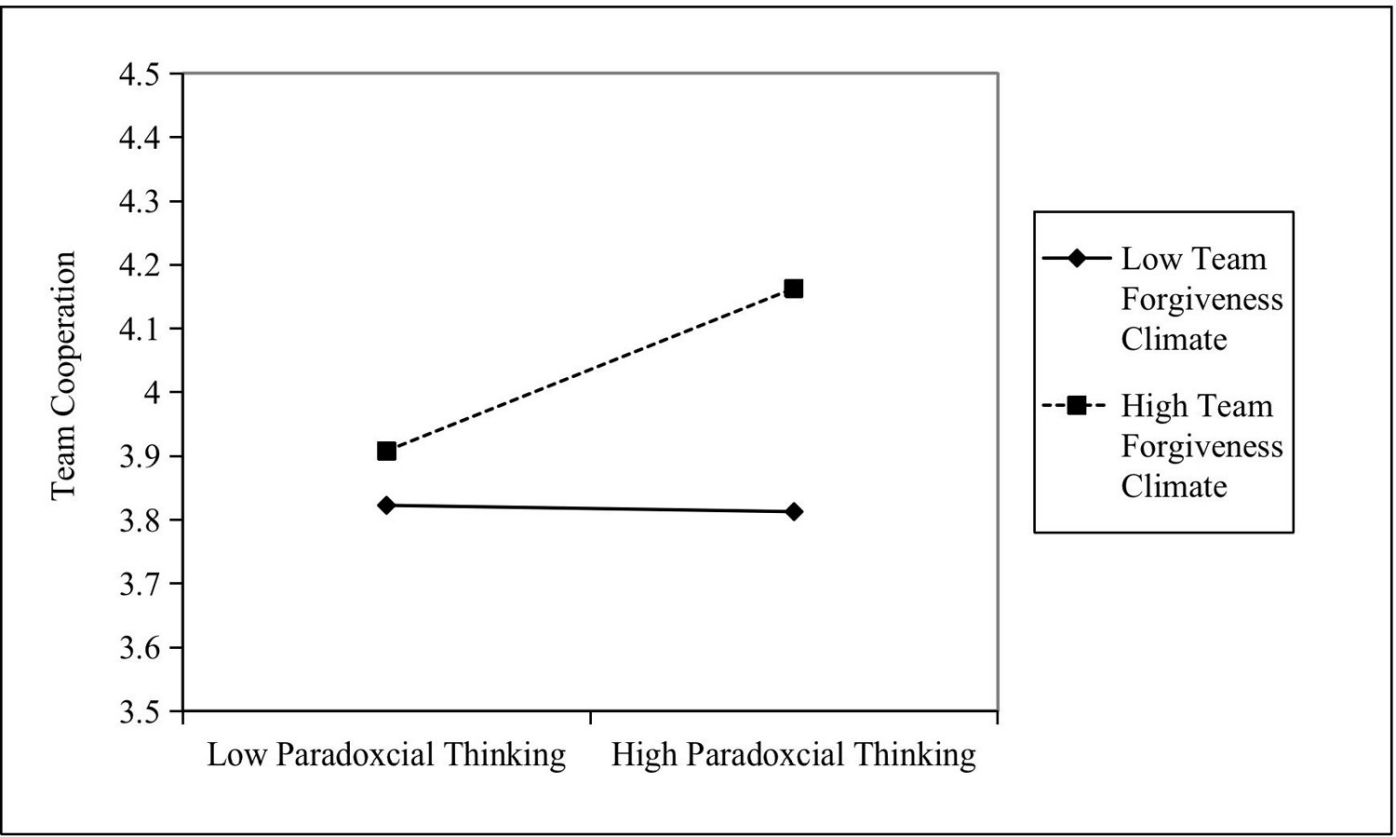
The impact of the interaction between paradoxical thinking and forgiveness climate on team cooperation.

Analysis of Hypotheses 3 and 4: The results of Model 3 in Table 3 indicate that the interaction between the paradoxical thinking of the leader and team forgiveness climate is significant after controlling the natural attributes of the leader and team size (B = 0.214, SE = 0.078, P < 0.01). This interaction term produces ΔR^2^ = 0.266 (P < 0.001) based on the control and manipulated variables. Therefore, the team forgiveness climate moderates the relationship between the paradoxical thinking of leaders and team cooperation. In order to explain this significant moderating effect, the method proposed by Aiken and West [64] was used to adjust the levels of moderating variables to determine the mean (plus or minus) by one standard deviation (±1SD). As shown in Fig 2, the result indicates that the high team forgiveness climate means that the positive influence of the paradoxical thinking of leaders on team cooperation is relatively strong. Therefore, Hypothesis 3 holds.

In order to further test the moderated mediating effect, this paper uses the Bootstrap method to test the moderated mediating model based on existing research. The specific analysis results are shown in Table 5.

**Table 5:** Bootstrap test of moderated mediating effect.

Variables	Mediating variables	Moderating variables	Indirect effect	Standard deviation	95% confidence interval	
					Lower confidence limit	Higher confidence limit
Paradoxical thinking		Low(-1SD)	-.005	0.044	-.115	0.061
	Team cooperation	Forgiveness climate			-	
		High(+1SD)	0.135	0.070	0.00	0.277

Note: The sample size of Bootstrap is N = 5000.

The indirect effect value in a high forgiveness climate is 0.135, with its figure [.006, .277] in the 95% confidence interval. However, it is -0.005 in a low forgiveness climate, with its figure [-.115, .061] in the 95% confidence interval. Thus, the forgiveness climate only moderates the mediating role of team cooperation in the relationship between the paradoxical thinking of leaders and team voice behavior to a relatively high degree. Therefore, Hypothesis 4 holds.

## 5. Research conclusions and discussions

### 5.1 Research conclusion

Based on the social exchange theory, this study examines the mediating role of team cooperation between the paradoxical thinking of leaders and team voice behavior from leaders’ thinking characteristics. The moderating effect of a team forgiveness climate is investigated to reveal the influence mechanism of leaders’ paradoxical thinking on team voice behavior. Based on the "leader-employee" matching questionnaire of 477 employees in 101 teams, conclusions are drawn as follows: 1. The paradoxical thinking of leaders predicted team voice behavior; 2. Team cooperation mediated the relationship between the paradoxical thinking of leaders and team voice behavior; 3.Moreover, the positive relationship between the paradoxical thinking of leaders and team cooperation is stronger, when the forgiveness climate is stronger. In contrast, the positive relationship between the paradoxical thinking of leaders and team cooperation is weaker, when the forgiveness climate is lower; 4. Team forgiveness climate also exerts a positive role in moderating the indirect effect of the paradoxical thinking of leaders on team voice behavior through team cooperation: in a stronger team forgiveness climate, the indirect effect is stronger.

### 5.2 Theoretical significance

First, this research starts with the characteristics of the paradoxical thinking of leaders and expands the antecedent variables of team voice behavior from the perspective that personal thinking characteristics of leaders influence team. The paradoxical thinking of leaders has a positive predictive effect on the team voice behavior. Although scholars have done relevant studies on paradoxical leaders [17,65], most studies are based on the impact of paradoxical leaders on employee voice. This research can enrich the research concerning the influence of the paradoxical thinking of leaders on team voice from the team level.

Second, team cooperation plays a mediating and explanatory role in the influence of the paradoxical thinking of leaders on team voice. From the perspective of the influence of leaders’ thinking characteristics on team members and the communication and cooperation of team members due to leaders’ acceptance and understanding, this study reveals the effect path of the paradoxical thinking characteristics of leaders on their team voice behavior. Additionally, whether team cooperation is the bridge between the paradoxical thinking of leaders and team voice behavior is tested.

Finally, this study verifies that the team forgiveness climate positively moderates the relationship between the paradoxical thinking of leaders and team cooperation. Moreover, the mediating role of team cooperation in the influence of the paradoxical thinking of leaders on team voice is verified. When the forgiveness climate releases a signal similar to embrace failure, team members conduct more communication and cooperation to repay the team’s forgiveness, resulting in more proactive behaviors and positive expressions. This conclusion supports the mutually beneficial view in social exchange theory. In a team forgiveness climate, team members are more willing to communicate and cooperate and are more proactive in expressing their opinions, with the team showing more voice behaviors. This paper expands the boundary condition research on the relationship between the paradoxical thinking of leaders and team voice behavior. It also reveals whether the contextual factors of team forgiveness climate influence the effect path of the paradoxical thinking of leaders on the team voice behavior through team cooperation. That is how this work can enrich the theoretical research of team voice formation.

### 5.3 Practical significance

This study provides the following inspiration for companies to stimulate team voice behavior.

First, the cultivation of the paradoxical thinking that shapes managers: 1. The correct identification of the thinking characteristics of leaders to guide them to maintain moderate paradoxical thinking. 2. The cultivation of managers’ paradoxical thinking needs to focus on their training to develop holistic mindsets. Their understanding of two aspects of contradictions in the team should be enhanced, and two extremes of contradictions should be integrated, to promote a more proactive expression of teams.

Second, the guidance of team cooperation: Since team cooperation plays a mediating role in the relationship between the paradoxical thinking of leaders and team voice behavior, business managers should guide and encourage employees to complete team cooperation goals cooperatively in daily work. Additionally, managers also should provide resources that promote team cooperation and create a team culture atmosphere of win-win cooperation.

Finally, the creation of a team forgiveness climate: Team forgiveness climate positively moderates the mediating role of team cooperation in the influence of the paradoxical thinking of leaders on team voice behavior. Thus, the enterprise should establish a team forgiveness climate and dispel concerns of team members on the possible negative effects of voice behavior. In addition, in a team with a forgiveness climate, the paradoxical thinking of leaders is more likely to positively predict the team voice behavior, which is conducive to exerting the role models of leaders. Enterprise managers should pay attention to the construction of team culture. They can encourage the team to offer suggestions by establishing a good relationship with employees, understanding their work, and allowing different opinions.

## 6. Research limitations and future research directions

This research is exploratory research on team voice with Chinese mainland corporate teams as the object. Although the research has achieved some valuable conclusions, the following limitations exist. First, although this study adopts survey data from multiple sources at multiple stages and time points, the team voice behavior may fail to conduct an accurate evaluation because team voice is evaluated by the leaders of team members. Therefore, the following research is suggested to adopt the multi-sources (team members—team leaders—leaders of team leaders) and multi-stage investigation methods. Moreover, the outcome variable of team voice behavior should be filled out by leaders of team leaders. Second, the too-much-of-a-good-thing (TMGT) effect [66] in paradoxical thinking is not considered. Studies have found that excessive paradoxical thinking can cause individuals to spend much time integrating contradictory elements and neglect non-logical thinking such as imagination and intuition [67]. The paradoxical thinking of leaders is too low to understand and integrate different knowledge and ideas, thus reducing the voice behavior of the team [67,68]. Therefore, the organization should be concerned about the paradoxical thinking level of the leader at a medium level, which is conducive to the positive development of the organization. Similarly, if the forgiveness climate is too low, team members will feel harsh and severe, which makes the social exchange relationship worse and the guidance and support provided by the team less, affecting team cooperation [69,70]. On the contrary, when the team has a high forgiveness climate, it is easy to be interpreted as not completing tasks and not being responsible, thus affecting the development of the team and the organization [71,72]. Therefore, the forgiveness climate is conducive to the development of the organization and the team in the middle degree, and the forgiveness climate in the high and low degree has a certain negative impact on the staff and the team [73]. According to the above, our team suggests that in future research, we should consider the too-much-of-a-good-thing effect of paradoxical thinking and forgiveness climate, which may provide a new perspective for future research [68]. Of course, future research should also consider the pros and cons and the degree of paradoxical thinking that has the greatest positive effect. Finally, the data in this study are collected from Shanghai, Guangdong, Zhejiang, Jiangsu, Guizhou and Sichuan province in Chinese mainland. Therefore, whether the relationship between the variables found in this study is universal for companies in other areas needs more research.

**Note:** A sample of the questionnaire to provide complete transparency to the context and framing that led to the generated data.

## Supporting information

S1 File Data to support the research model. (PDF)
