# Supplementary material for: In a team forgiveness climate, the influence of paradoxical thinking of leaders on the team voice behavior: Mediated by team cooperation
Source: PLoS One. 2022 Mar 15;17(3):e0265018. doi: 10.1371/journal.pone.0265018 (PMC8923504; doi:10.1371/journal.pone.0265018)
Supplement: S1 File — (PDF) [file pone.0265018.s001.pdf]

idd	sgen_mea	sage_mea	sedu_mea	year_mea	teamsize_	paradox_	forge_me	coope_me	tvoice_me
11	1.00	45.00	18.00	13.00	10.00	4.11	4.25	4.38	4.00
21	0.00	45.00	18.00	18.00	4.00	4.11	4.25	4.19	3.67
31	0.00	35.00	18.00	10.00	6.00	3.89	3.50	4.00	4.00
41	1.00	36.00	18.00	1.00	15.00	4.00	4.75	4.20	4.83
51	0.00	42.00	18.00	20.00	3.00	5.00	3.50	4.08	3.50
61	1.00	40.00	18.00	12.00	9.00	3.67	4.00	4.25	4.83
71	0.00	34.00	18.00	7.00	7.00	4.22	5.00	4.21	4.67
81	1.00	38.00	18.00	12.00	10.00	5.00	5.00	4.08	4.83
91	0.00	35.00	18.00	10.00	6.00	4.33	5.00	4.25	4.00
101	0.00	56.00	18.00	20.00	10.00	4.11	4.00	4.10	4.17
111	1.00	40.00	16.00	10.00	8.00	3.11	3.00	4.04	3.67
121	1.00	39.00	16.00	3.00	6.00	3.89	3.25	3.83	3.67
131	1.00	46.00	16.00	14.00	10.00	4.33	4.50	4.04	3.33
141	1.00	40.00	16.00	9.00	6.00	3.78	4.25	4.13	4.33
151	0.00	42.00	18.00	9.00	7.00	3.56	4.00	3.75	4.33
161	0.00	38.00	15.00	4.00	6.00	3.89	5.00	4.38	4.00
171	0.00	48.00	16.00	20.00	6.00	3.89	5.00	4.25	4.67
181	1.00	44.00	18.00	16.00	6.00	3.56	4.00	3.88	3.83
191	1.00	41.00	18.00	10.00	20.00	4.22	5.00	4.33	4.67
201	1.00	34.00	16.00	3.00	10.00	3.78	3.75	3.83	4.33
211	1.00	34.00	16.00	3.00	10.00	3.78	3.75	3.75	4.33
221	0.00	41.00	18.00	3.00	4.00	3.78	5.00	3.75	3.50
231	0.00	39.00	18.00	11.00	20.00	4.33	3.75	4.50	4.50
241	1.00	40.00	18.00	10.00	4.00	3.33	5.00	3.81	4.83
251	1.00	36.00	18.00	5.00	9.00	3.22	4.75	4.30	4.33
261	0.00	39.00	18.00	10.00	10.00	5.00	4.75	3.92	3.33
271	0.00	38.00	16.00	5.00	10.00	4.22	4.00	3.75	4.00
281	1.00	36.00	15.00	6.00	6.00	3.56	5.00	4.13	4.00
291	0.00	49.00	18.00	12.00	11.00	4.78	5.00	4.29	4.83
301	0.00	38.00	16.00	9.00	8.00	4.00	4.75	4.00	4.00
311	0.00	46.00	15.00	13.00	8.00	3.89	4.00	4.08	3.83
321	0.00	51.00	16.00	10.00	6.00	4.11	4.25	4.17	4.33
331	1.00	38.00	16.00	10.00	5.00	4.33	5.00	4.15	4.17
341	1.00	35.00	18.00	5.00	8.00	5.00	5.00	5.00	5.00
351	1.00	35.00	18.00	4.00	10.00	4.44	5.00	3.88	4.00
361	1.00	33.00	18.00	3.00	6.00	3.67	5.00	3.83	4.00
371	0.00	40.00	18.00	6.00	9.00	4.00	4.75	4.21	4.00
381	0.00	56.00	18.00	20.00	12.00	3.67	4.00	4.33	4.17
391	0.00	49.00	16.00	17.00	6.00	4.00	4.00	4.21	4.00
401	0.00	47.00	16.00	10.00	8.00	3.56	4.00	4.33	3.83
411	0.00	45.00	16.00	14.00	4.00	3.33	4.00	4.25	3.83
421	1.00	50.00	18.00	10.00	10.00	3.00	4.50	3.83	4.50
431	0.00	53.00	16.00	25.00	6.00	4.33	4.50	4.38	3.33
441	1.00	36.00	18.00	6.00	6.00	3.89	3.75	3.67	5.00
451	0.00	46.00	16.00	11.00	6.00	4.00	5.00	4.55	4.00
461	0.00	45.00	16.00	16.00	9.00	4.44	4.25	4.42	3.83
471	0.00	38.00	18.00	15.00	7.00	3.56	3.75	4.29	3.83
481	1.00	34.00	16.00	5.00	4.00	3.22	3.75	3.58	5.00
491	0.00	39.00	16.00	8.00	8.00	3.22	5.00	4.00	4.33
501	0.00	47.00	16.00	12.00	10.00	4.89	4.25	4.33	5.00
511	1.00	40.00	16.00	8.00	6.00	3.78	4.00	3.75	3.67
521	1.00	40.00	16.00	12.00	4.00	4.11	4.75	3.81	3.83
531	1.00	33.00	16.00	8.00	8.00	4.11	4.75	4.33	4.33

541	1.00	31.00	16.00	8.00	4.00	3.56	3.00	3.58	3.17
551	0.00	46.00	18.00	15.00	9.00	4.56	4.00	3.96	4.00
561	0.00	45.00	18.00	9.00	8.00	3.44	4.00	4.15	4.17
571	0.00	46.00	16.00	17.00	4.00	4.33	4.00	4.25	3.83
581	0.00	29.00	18.00	1.50	4.00	3.89	4.00	4.19	3.83
591	0.00	39.00	16.00	10.00	11.00	3.89	3.75	3.88	3.67
601	0.00	29.00	15.00	5.00	5.00	4.44	5.00	4.31	4.67
611	0.00	28.00	15.00	5.00	4.00	3.22	3.50	3.94	4.00
621	0.00	34.00	15.00	9.00	5.00	3.56	3.75	4.13	3.33
631	0.00	27.00	15.00	5.00	15.00	5.00	5.00	4.56	4.67
641	0.00	50.00	16.00	8.00	5.00	3.67	3.75	4.00	4.00
651	0.00	55.00	16.00	30.00	6.00	3.33	3.25	3.94	3.67
661	1.00	50.00	15.00	28.00	4.00	4.00	4.00	3.83	5.00
671	0.00	52.00	18.00	15.00	10.00	3.33	4.25	4.38	4.17
681	1.00	43.00	21.00	8.00	15.00	3.56	4.00	4.00	4.33
691	1.00	30.00	16.00	1.00	6.00	3.33	3.75	4.13	4.50
701	0.00	42.00	18.00	10.00	15.00	4.11	4.75	4.33	4.67
711	1.00	42.00	15.00	16.00	6.00	4.11	3.75	4.08	3.83
721	1.00	30.00	16.00	1.00	6.00	3.33	3.75	4.13	4.50
731	0.00	42.00	18.00	10.00	15.00	4.11	4.75	4.33	4.67
741	1.00	42.00	15.00	16.00	6.00	4.11	3.75	4.08	3.83
751	0.00	27.00	15.00	5.00	15.00	5.00	5.00	4.56	4.67
761	0.00	27.00	15.00	5.00	15.00	5.00	5.00	4.56	4.67
771	0.00	50.00	16.00	8.00	5.00	3.67	3.75	4.00	4.00
781	0.00	50.00	16.00	8.00	5.00	3.67	3.75	4.00	4.00
791	0.00	50.00	16.00	8.00	5.00	3.67	3.75	4.00	4.00
801	0.00	55.00	16.00	30.00	6.00	3.33	3.25	3.94	3.67
811	1.00	50.00	15.00	28.00	4.00	4.00	4.00	3.83	5.00
821	0.00	52.00	18.00	15.00	10.00	3.33	4.25	4.38	4.17
831	1.00	43.00	21.00	8.00	15.00	3.56	4.00	4.00	4.33
841	0.00	39.00	16.00	10.00	11.00	3.89	3.75	3.88	3.67
851	0.00	39.00	16.00	10.00	11.00	3.89	3.75	3.88	3.67
861	0.00	39.00	16.00	10.00	11.00	3.89	3.75	3.88	3.67
871	0.00	29.00	15.00	5.00	5.00	4.44	5.00	4.31	4.67
881	0.00	28.00	15.00	5.00	4.00	3.22	3.50	3.94	4.00
891	0.00	34.00	15.00	9.00	5.00	3.56	3.75	4.13	3.33
901	0.00	27.00	15.00	5.00	14.00	5.00	5.00	4.56	4.67
911	0.00	27.00	15.00	5.00	15.00	5.00	5.00	4.56	4.67
921	0.00	27.00	15.00	5.00	15.00	5.00	5.00	4.56	4.67
931	1.00	35.00	18.00	5.00	8.00	5.00	5.00	5.00	5.00
941	1.00	35.00	18.00	5.00	8.00	5.00	5.00	5.00	5.00
951	1.00	40.00	16.00	12.00	4.00	4.11	4.75	3.81	3.83
961	1.00	33.00	16.00	8.00	8.00	4.11	4.75	4.33	4.33
971	1.00	31.00	16.00	8.00	4.00	3.56	3.00	3.58	3.17
981	0.00	46.00	18.00	15.00	9.00	4.56	4.00	3.96	4.00
991	0.00	45.00	18.00	9.00	8.00	3.44	4.00	4.15	4.17
1001	0.00	46.00	16.00	17.00	4.00	4.33	4.00	4.25	3.83
1011	0.00	29.00	18.00	1.50	4.00	3.89	4.00	4.19	3.83

ean
